# RNA-Based Therapies for Hypercholesterolemia and Coronary Artery Disease

**DOI:** 10.7759/cureus.103877

**Published:** 2026-02-18

**Authors:** Maynor Jose Lopez Mendoza, Nicolle Contreras Figueroa, María Jennifer Valle Mena, Asdrubal Ulloa, Jeilyn Jiron Vindas, Maria Antonieta Salazar Estrada

**Affiliations:** 1 Anesthesiology and Perioperative Medicine, Hospital de las Mujeres "Adolfo Carit Eva" (HOMACE) Caja Costarricense de Seguro Social (CCSS), San Jose, CRI; 2 General Medicine, Costa Rican Social Security System (CCSS), San Jose, CRI; 3 General Medicine, Área de Salud Upala, Alajuela, CRI; 4 Gynecologic Oncology, Hospital de las Mujeres "Adolfo Carit Eva" (HOMACE) Caja Costarricense de Seguro Social (CCSS), San Jose, CRI; 5 Obstetrics and Gynecology, Hospital de las Mujeres "Adolfo Carit Eva" (HOMACE) Caja Costarricense de Seguro Social (CCSS), San Jose, CRI; 6 Emergency, Hospital Los Chiles, Alajuela, CRI

**Keywords:** antisense oligonucleotides, cardiovascular risk, hypercholesterolemia, pcsk9 inhibition, rna therapies, sirna

## Abstract

RNA-based therapies have emerged as a transformative approach in the management of hypercholesterolemia and coronary artery disease by directly targeting molecular pathways involved in lipid regulation. These treatments focus on silencing key genes such as PCSK9, ANGPTL3, ApoB, and Lp(a), achieving substantial reductions in low-density lipoprotein cholesterol (LDL-C), triglycerides, and other atherogenic lipoproteins. Small interfering RNA (siRNA) and antisense oligonucleotides (ASOs) provide highly specific post-transcriptional gene suppression, while advances in chemical stabilization and GalNAc conjugation have enhanced hepatocyte delivery and prolonged therapeutic action. Approved agents such as inclisiran demonstrate sustained LDL-C reductions of approximately 50% with only two to three injections annually, improving adherence and offering an alternative for patients intolerant to statins or unable to reach lipid targets with conventional therapy. Pelacarsen and other emerging antisense therapies show promise for reducing lipoprotein(a), an independent cardiovascular risk factor, while siRNAs targeting ANGPTL3 offer prolonged lipid-lowering effects beyond those achieved with monoclonal antibodies. Despite these advantages, challenges remain. Hepatic safety concerns have halted the development of some agents, such as vupanorsen, and long-term cardiovascular outcome data for several therapies, including inclisiran, are still in development. Cost and accessibility also limit broad adoption, emphasizing the need for cost-effective strategies and long-term surveillance. Nevertheless, current evidence supports the integration of RNA-based therapies into modern lipid-lowering algorithms, particularly for high-risk patients, while ongoing research continues to refine delivery systems, enhance safety, and expand therapeutic indications.

## Introduction and background

Hypercholesterolemia is a major risk factor for atherosclerotic cardiovascular disease (ASCVD), affecting a significant portion of the adult population worldwide [1]. Its presence is commonly associated with elevated concentrations of cholesterol, triglycerides, and lipoproteins, alterations that not only promote atherogenesis but also contribute to related metabolic conditions such as type 2 diabetes and non-alcoholic fatty liver disease [2].

Despite the availability of multiple lipid-lowering agents, conventional therapies present important limitations. Statins remain the first-line treatment, but their long-term use may be restricted by intolerable adverse effects, reducing adherence and therapeutic continuity. Similarly, monoclonal antibodies such as evolocumab and alirocumab achieve significant reductions in low-density lipoprotein cholesterol (LDL-C) levels; however, their short half-life and elevated cost pose barriers to sustained use, often leading to non-compliance [2]. Beyond adherence issues, traditional pharmacologic strategies frequently fail to modulate metabolic pathways with the precision required for optimal lipid regulation [3].

In this context, RNA-based therapies have emerged as a promising alternative due to their capacity for tissue-specific targeting and highly controlled modulation of gene expression. Through platforms such as antisense oligonucleotides (ASOs) and small interfering RNA (siRNA), these approaches effectively suppress the expression of proteins for which no small molecule inhibitors are available, thereby expanding the therapeutic landscape for dyslipidemia. Additionally, their extended dosing intervals, ranging from weekly to biannual administration, offer a practical advantage by reducing pill burden and simplifying treatment schedules, which may further enhance adherence and therapeutic outcomes [3].

The general objective of this review is to provide a comprehensive overview of RNA-based therapies for hypercholesterolemia and coronary artery disease, emphasizing their potential to address the limitations associated with conventional treatment strategies. In alignment with this aim, the specific objectives focus on examining the mechanisms through which these therapies exert their effects, evaluating their clinical applications, and assessing their safety profiles, thereby establishing a clear understanding of their therapeutic relevance. Additionally, the review seeks to discuss the role of RNA-based interventions within the broader framework of precision medicine and individualized care, as highlighted in recent literature [4,5].

The objective of this article is to analyze the current evidence on RNA-based therapies for the management of hypercholesterolemia and their potential impact on reducing the burden of coronary artery disease.

## Review

Methods

The narrative review was developed using a focused yet flexible search strategy to generate a comprehensive synthesis of current evidence on RNA-based therapies for hypercholesterolemia and coronary artery disease. To ensure adequate breadth and depth of the literature, the search was conducted across three major biomedical databases (PubMed, Scopus, and ScienceDirect), all of which offer extensive international coverage of peer-reviewed medical and scientific publications.

The search strategy restricted the selection of articles to those published within the past five years (2020-2025) in order to capture the most recent and clinically relevant findings. Studies available in English and Spanish were included to ensure accuracy in interpretation and comprehension. A combination of MeSH terms and free-text descriptors was employed, incorporating keywords such as RNA therapies, hypercholesterolemia, PCSK9 inhibition, siRNA, antisense oligonucleotides, and cardiovascular risk. Boolean operators were applied to refine the search and retrieve studies addressing therapeutic mechanisms, clinical efficacy, safety, and translational applicability within cardiovascular medicine.

Given that rigid systematic criteria may inadvertently exclude contemporary or clinically meaningful studies, a flexible selection approach was adopted. Articles were chosen based on relevance, quality, and applicability to clinical practice rather than strictly on study design. Eligible literature included original research articles, systematic reviews, meta-analyses, and expert consensus statements published in reputable scientific journals or by recognized medical societies. Studies that were duplicated, lacked clinical relevance, or did not report outcomes pertinent to the population of interest were excluded.

Publications that, despite scientific merit, did not provide clinically transferable information were also discarded. This included laboratory-based or animal-only studies without a clear link to patient care, research conducted in populations not comparable to those with hypercholesterolemia or coronary artery disease, or studies whose findings could not be meaningfully integrated into therapeutic decision-making. Only evidence offering practical and clinically applicable insights into RNA-based therapeutic approaches was retained.

The final body of literature included observational studies, clinical trials, narrative and systematic reviews, experimental research relevant to translational mechanisms, and position papers addressing emerging therapeutic modalities. This allowed for a broad and critical perspective on the evolving role of RNA-based therapies in lipid management and cardiovascular risk reduction.

Additionally, artificial intelligence (AI) tools were used to optimize the legibility and style of the written content and to ensure that the text remained free of grammatical, spelling, punctuation, and tone-related errors.

The authors used OpenAI’s artificial intelligence (AI) tool to assist in the structural organization and linguistic refinement of the manuscript. While AI-assisted editing contributed to clarity and cohesion, all interpretative decisions, content validation, and scientific judgment were performed exclusively by the authors to preserve the academic and clinical rigor of the work.

Molecular basis of lipid regulation

The molecular basis of lipid regulation involves several key targets that have become central to the development of RNA-based therapies. PCSK9 plays a critical role in the degradation of LDL receptors, and its inhibition produces significant reductions in LDL-C. In this context, RNA-based approaches such as small interfering RNAs have demonstrated reductions of up to 97% in PCSK9 levels, leading to a corresponding 67% decrease in LDL-C. Similarly, ANGPTL3 has emerged as an important regulator of triglyceride and cholesterol metabolism. Targeting this protein with RNA interference therapeutics, such as zodasiran, has resulted in substantial reductions in triglycerides and LDL-C, with particular benefit for patients who have limited LDL receptor function [6]. Other relevant targets include ApoB and Lp(a), for which RNA-directed treatments have shown efficacy in lowering circulating concentrations, thereby contributing to cardiovascular risk reduction [7].

**Figure 1:**
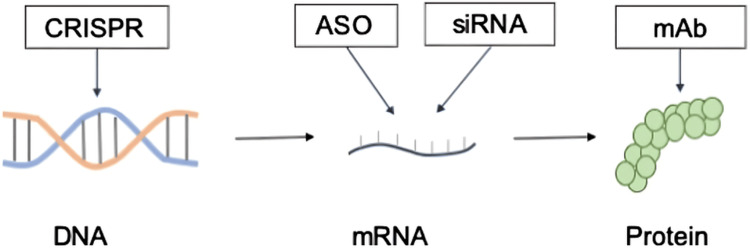
Molecular targets of novel lipid-lowering biologics include targeting of proteins by mAb, mRNA by ASO, and siRNA therapeutics CRISPR gene-editing technology that works at the DNA level is also being developed. mAb: monoclonal antibodies, mRNA: messenger RNA, ASO: antisense oligonucleotide, siRNA: small interfering RNA Figure reproduced from Mäkinen et al., licensed under Creative Commons Attribution (CC BY) [8]

To contextualize these therapeutic strategies, the different molecular levels at which lipid-lowering biologics act can be visualized in Figure 1. These approaches target DNA through gene-editing platforms such as CRISPR, messenger RNA (mRNA) through antisense oligonucleotides and small interfering RNAs, and proteins through monoclonal antibodies, illustrating the breadth of mechanisms currently employed in lipid regulation.

The biological rationale for using RNA modulation in lipid disorders lies in its ability to silence genes involved in cholesterol and lipoprotein metabolism. Through mechanisms such as RNA interference, these therapies provide a targeted approach to decreasing lipid levels by directly reducing the expression of proteins implicated in dyslipidemia [9]. This molecular precision also underpins several therapeutic advantages. Compared with conventional lipid-lowering treatments, RNA-based therapies offer longer-lasting effects and reduced dosing frequency, features that may improve adherence and overall treatment effectiveness [6].

Although these therapies show considerable promise, important challenges persist. Long-term safety data remain limited, and the development of cost-effective strategies is necessary to enable wider access. Furthermore, the extent to which their efficacy translates across diverse populations and their ultimate effect on clinical outcomes require confirmation through large-scale trials [6,9].

Biological and technological foundations of RNA-based therapies

Small interfering RNA represents a highly specific approach to gene silencing by incorporating into the RNA-induced silencing complex, which subsequently degrades complementary messenger RNA and produces post-transcriptional suppression of gene expression. Through this mechanism, siRNA enables precise downregulation of selected genes involved in lipid regulation [10,11]. Because native siRNAs are prone to rapid degradation and exhibit limited tissue penetration, they undergo chemical stabilization and are frequently conjugated with N-acetylgalactosamine to enhance hepatocyte uptake. This strategy has been successfully implemented in several FDA-approved siRNA therapeutics, including patisiran and inclisiran, both of which target genes expressed predominantly in the liver [10,12,13].

Antisense oligonucleotides constitute another major RNA-based platform. These molecules bind directly to target RNA sequences and promote RNase H-mediated degradation of the complementary strand, thereby reducing the expression of the corresponding gene. This mechanism is also applicable to the modulation of RNA splicing and the suppression of pathogenic protein production [14]. Advances in ASO chemistry have produced second- and third-generation molecules incorporating modifications such as 2’-O-methoxyethyl substitutions and locked nucleic acids, which increase binding affinity and stability. As with siRNA, GalNAc conjugation has become a central strategy to enhance liver-specific delivery [14,15].

**Figure 2:**
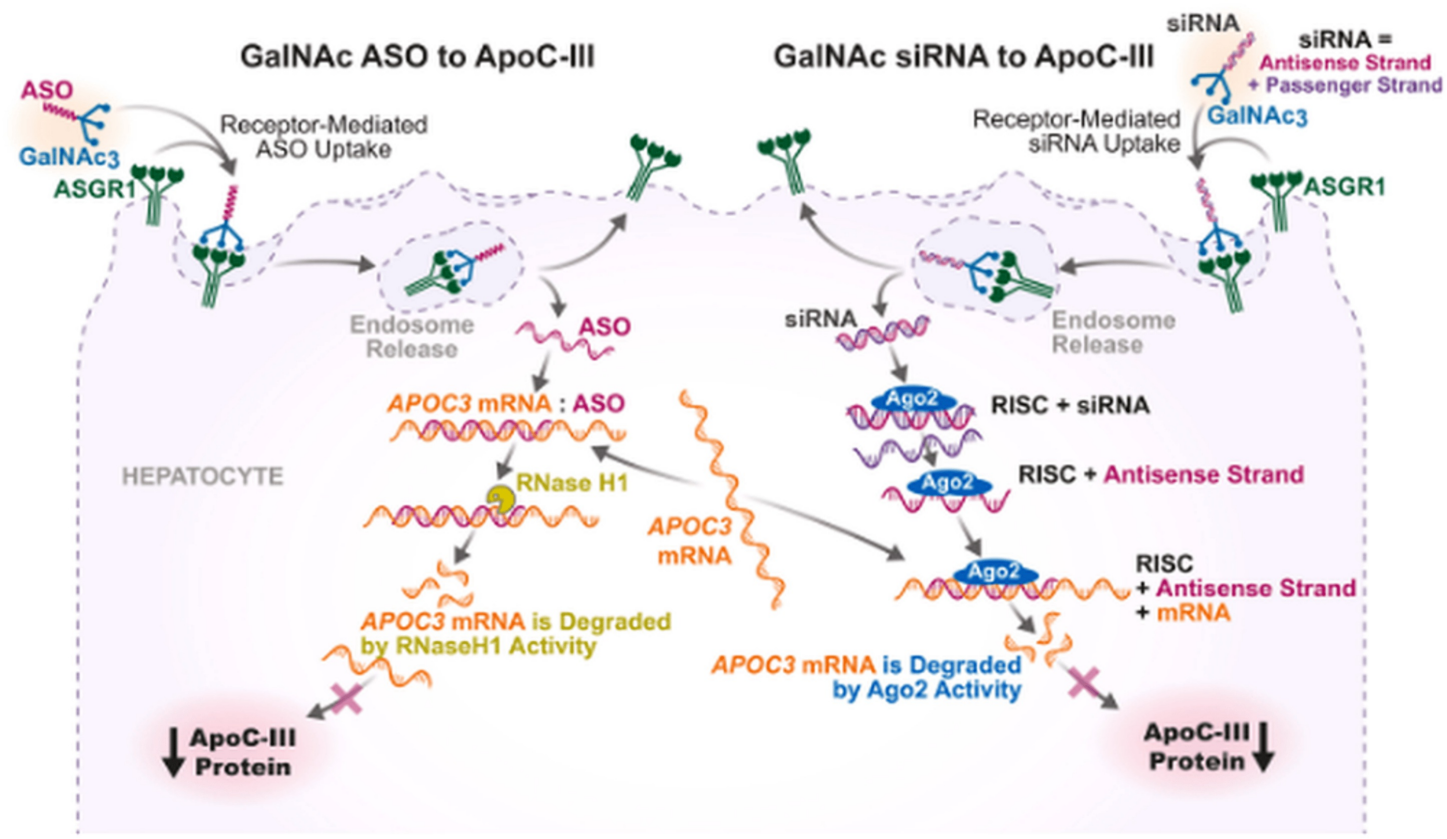
Mechanisms of action of GalNAc-conjugated antisense oligonucleotides and siRNA targeting ApoC-III Ago2: argonaute 2, ApoC-III: apolipoprotein C-III, ASGR1: asialoglycoprotein receptor 1, ASO: antisense oligonucleotide, GalNAc₃: triantennary N-acetylgalactosamine, RISC: RNA-induced silencing complex, RNase H1: ribonuclease H1, siRNA: small interfering RNA Figure reproduced from Brinton et al., licensed under CC BY-NC 4.0 [16]

The following schematic (Figure 2) summarizes the hepatocyte-specific mechanisms of action of GalNAc-conjugated antisense oligonucleotides and siRNA, illustrating their differential uptake pathways and gene-silencing processes.

Therapeutic messenger RNA represents a different modality, relying on the administration of exogenous mRNA to induce cellular production of protective proteins. This approach is currently used in vaccine development and is being investigated for broader protein replacement applications [17]. However, its use in cardiovascular disease remains limited due to challenges related to delivery efficiency and mRNA stability within the circulatory system, issues that necessitate further refinement before reliable clinical application can be achieved [18].

CRISPR-based RNA-guided gene editing offers yet another innovative approach by using RNA sequences to direct gene-editing enzymes toward specific DNA targets. Techniques such as base editing and prime editing enable precise modification of hepatocyte DNA, providing a potential means to regulate lipid metabolism at the genomic level. These applications may eventually expand therapeutic options for lipid disorders and related metabolic conditions, although their clinical utility is still under investigation [18].

Approved or late-stage RNA therapies for hypercholesterolemia

Inclisiran is a small interfering RNA therapy specifically designed to target PCSK9 messenger RNA, thereby inhibiting its hepatic synthesis and promoting increased recycling of LDL receptors. This mechanism results in a marked reduction in circulating LDL cholesterol levels. Its pharmacokinetic profile supports an infrequent dosing regimen: an initial subcutaneous dose, a second dose at three months, and subsequent administrations every six months. This twice-yearly schedule is intended to improve adherence by reducing the treatment burden associated with more frequent dosing. Evidence from major clinical trials, including ORION-9, ORION-10, and ORION-11, has demonstrated sustained LDL-C reductions of approximately 50% compared with placebo. These trials included participants with heterozygous familial hypercholesterolemia and established cardiovascular disease, confirming the therapy’s efficacy across high-risk populations. Inclisiran also exhibits a favorable safety profile, with mild injection site reactions being the most frequently reported adverse events, and its biannual dosing strategy is expected to facilitate greater long-term adherence [19-23].

Pelacarsen, an antisense oligonucleotide targeting lipoprotein(a), addresses the clinical need to reduce elevated Lp(a), which is a recognized and independent risk factor for cardiovascular disease. Lowering Lp(a) levels is particularly important for patients with high cardiovascular risk who do not adequately respond to conventional therapies. The HORIZON trial is evaluating the efficacy and safety of pelacarsen in reducing Lp(a) concentrations and associated cardiovascular outcomes. Interim findings indicate that pelacarsen produces substantial reductions in Lp(a) levels and maintains a safety profile consistent with other antisense oligonucleotide therapies [19].

Vupanorsen targets ANGPTL3, a key regulator of lipid metabolism, with the aim of lowering LDL-C and triglycerides while potentially increasing HDL levels. Despite its promising mechanism of action, clinical development was halted due to hepatic safety concerns, underscoring the importance of close monitoring of liver function in patients receiving RNA-based treatments [19].

Mipomersen is an antisense oligonucleotide designed for individuals with homozygous familial hypercholesterolemia, a condition characterized by severely elevated cholesterol levels from early life. However, its clinical use is constrained by the risk of hepatic toxicity, necessitating ongoing surveillance of liver enzymes during therapy. These limitations have affected its broader adoption in clinical practice [19,21].

Emerging RNA therapies in development

SiRNA therapies targeting ANGPTL3, such as ARO-ANG3, aim to reduce plasma triglycerides and LDL-C by silencing ANGPTL3 mRNA. This mechanism distinguishes them from monoclonal antibodies such as evinacumab, which also inhibit ANGPTL3 but act at the protein level. Because siRNA therapies suppress gene expression directly, they produce a more sustained effect, resulting in prolonged lipid lowering compared with monoclonal antibody-based treatments [24,25]. Their long-acting profile is further supported by GalNAc conjugation, which enables efficient hepatocyte uptake and allows for infrequent dosing schedules. This extended duration of action not only improves adherence but may also reduce overall healthcare costs. Clinical data for ARO-ANG3 have shown stable and durable lipid reductions, highlighting its promise for long-term dyslipidemia management [13,24].

Advancements in antisense technology have led to the development of third-generation antisense oligonucleotides designed to improve stability, specificity, and pharmacokinetics. ASOs targeting PCSK9 and ApoC3 incorporate chemical modifications that enhance their overall performance while reducing off-target effects. These innovations facilitate more precise lipid lowering and better tolerability [9,26]. Early-phase clinical trials have demonstrated substantial reductions in LDL-C and triglycerides with these agents. Olezarsen, an ASO directed against ApoC3, has shown particular promise by lowering triglycerides without the thrombocytopenia observed with earlier ASOs such as volanesorsen [26].

Advanced delivery platforms further strengthen the therapeutic potential of RNA-based lipid-lowering agents. Next-generation lipid nanoparticles are being optimized to improve delivery efficiency and specificity to hepatocytes, thereby minimizing systemic exposure and reducing the risk of adverse effects. These delivery systems have shown the capacity to achieve liver-specific gene editing with limited off-target activity [27]. Tissue-specific conjugates, particularly GalNAc-based strategies, maintain a central role in directing both siRNAs and ASOs to the liver, enhancing therapeutic precision while limiting unintended distribution [13,28]. Additional efforts to reduce off-target toxicity include refining sequence specificity and optimizing delivery vehicles, both of which contribute to safer and more effective RNA-based interventions [27,28].

Clinical impact on coronary artery disease

Intensive LDL-C reduction has consistently been associated with meaningful decreases in cardiovascular risk. Numerous lipid-lowering therapies, including both statins and non-statin agents, demonstrate a linear relationship between the magnitude of LDL-C reduction and the decline in major adverse cardiovascular events. A meta-analysis found that intensive lipid-lowering strategies produced a 15% reduction in major adverse cardiovascular events and a 17% reduction in myocardial infarction, underscoring the clinical importance of sustained LDL-C lowering [29]. When comparing different therapeutic modalities, PCSK9 inhibitors such as evolocumab and alirocumab, along with the siRNA therapy inclisiran, have achieved substantial LDL-C reductions. Evolocumab produced a 61.09% decrease, alirocumab 46.35%, and inclisiran reductions ranging from 43.11% to 54.83%, effects similar to those observed with high-intensity statin therapy [19,30].

These benefits extend across high-risk subgroups. In patients with heterozygous familial hypercholesterolemia, inclisiran achieved a mean LDL-C reduction of 47.9%, reflecting its clinical utility in genetically driven dyslipidemias [19]. Current recommendations support the use of PCSK9 inhibitors for patients with familial hypercholesterolemia who remain above LDL-C targets despite optimized statin therapy [31]. For individuals who are statin-intolerant, non-statin therapies, including PCSK9 inhibitors and inclisiran, represent effective alternatives capable of delivering meaningful lipid reductions [32]. In the context of secondary prevention, PCSK9 inhibitors have demonstrated significant outcome improvements; they reduce major adverse cardiovascular events by 15% in patients with established atherosclerotic cardiovascular disease, and alirocumab has additionally been associated with reduced all-cause mortality [28,33].

Clinical guidelines further reflect this evidence. The American College of Cardiology/American Heart Association and the European Society of Cardiology recommend PCSK9 inhibitors for high-risk patients with atherosclerotic cardiovascular disease or familial hypercholesterolemia who fail to reach LDL-C goals despite maximally tolerated statins [34]. They are also endorsed for secondary prevention in patients with recent acute coronary syndrome, given their demonstrated cardiovascular benefit. However, evidence gaps remain, particularly regarding the long-term impact of inclisiran on cardiovascular outcomes, which is still under investigation [31]. Additionally, economic considerations and the need for subcutaneous administration continue to limit widespread clinical adoption [34].

Safety profile and immunological considerations

Injection site reactions are among the most commonly reported adverse events associated with inclisiran, given its subcutaneous route of administration. These reactions, which include mild redness, bruising, or swelling at the injection site, are generally transient and non-severe, and they rarely lead to treatment discontinuation [19,35]. Beyond local effects, hepatic safety is an important consideration for lipid-lowering therapies; however, long-term data indicate that inclisiran does not significantly increase hepatic events or liver enzyme elevations compared with placebo, supporting its favorable hepatic safety profile [23].

Off-target activity is another potential concern with RNA-based therapeutics. Inclisiran’s mechanism, which involves highly specific targeting of PCSK9 mRNA, minimizes unintended interactions with other genes, and clinical studies have not shown meaningful changes in platelet counts or immune cell populations, suggesting a low likelihood of off-target effects [33]. With respect to immunogenicity, RNA-based therapies can theoretically induce antidrug antibodies; however, inclisiran has shown a low incidence of such antibodies, and their presence has not been associated with increased adverse events or treatment interruption [27,36].

Given that inclisiran is a long-acting therapy, extended safety monitoring remains essential. Evidence from long-term studies such as ORION-3 demonstrates sustained LDL-C reductions and a consistent safety profile over four years of follow-up. Nonetheless, ongoing surveillance is necessary to identify any late-onset adverse effects and ensure continued patient safety [27].

Comparison with biological and conventional therapies

RNA-based therapies offer several advantages in lipid management, particularly through prolonged therapeutic action and reduced dosing frequency. Inclisiran requires only two to three injections annually, yet it provides sustained reductions in LDL-C, a feature that supports improved adherence when compared with daily statin use or the more frequent administration required for PCSK9 monoclonal antibodies [37,38]. High specificity is another benefit, as inclisiran precisely targets PCSK9 mRNA, producing LDL-C reductions of approximately 50% and enhancing LDL receptor activity in hepatocytes, effects comparable to those of high-intensity statins [19,39]. Additionally, siRNA production can be scaled efficiently, which may contribute to lower manufacturing costs over time [38].

Despite these advantages, RNA-based therapies also present limitations. Cost and accessibility remain significant concerns, as inclisiran is currently expensive relative to traditional lipid-lowering therapies, thereby affecting its cost-effectiveness. Although clinical evidence supports its favorable safety profile, continued monitoring is necessary because long-term safety data are still accumulating, and mild injection site reactions have been observed. Furthermore, ongoing studies are needed to clarify inclisiran’s long-term cardiovascular effects and its interaction with other lipid-lowering modalities, leaving important evidence gaps for future research [38,39].

Within contemporary lipid-lowering algorithms, inclisiran can be effectively integrated into existing treatment regimens. It may be used in combination with statins to help patients at high cardiovascular risk achieve LDL-C goals when statin therapy alone proves insufficient. The therapy’s infrequent dosing schedule also represents an important advantage, as improved adherence addresses a persistent barrier to achieving optimal LDL-C control with current therapies [19,36].

## Conclusions

RNA-based therapies represent a mechanistically precise and biologically robust strategy for lipid regulation by selectively targeting central mediators of cholesterol and lipoprotein metabolism, including PCSK9, ANGPTL3, ApoB, and lipoprotein(a). Gene silencing through these platforms produces substantial and sustained lipid reductions, supporting their role as effective alternatives or adjuncts to conventional lipid-lowering therapies. Clinical data, particularly for agents such as inclisiran and pelacarsen, demonstrate favorable efficacy and safety profiles in both clinical trials and real-world settings, with pronounced benefits in high-risk populations such as individuals with familial hypercholesterolemia or statin intolerance.

Despite these advances, several challenges limit broader clinical integration. High treatment costs, restricted accessibility, and the absence of definitive long-term cardiovascular outcome data remain significant barriers, while ongoing safety considerations necessitate continued hepatic and immunogenicity monitoring. Addressing these limitations through large-scale outcome trials, cost-effectiveness evaluations, and rigorous post-marketing surveillance will be essential to define the long-term role of RNA-based therapies in lipid management.
